# Phytochrome-dependent coordinate control of distinct aspects of nuclear and plastid gene expression during anterograde signaling and photomorphogenesis

**DOI:** 10.3389/fpls.2014.00171

**Published:** 2014-04-30

**Authors:** Sookyung Oh, Beronda L. Montgomery

**Affiliations:** ^1^Department of Energy—Plant Research Laboratory, Michigan State UniversityEast Lansing, MI, USA; ^2^Department of Biochemistry and Molecular Biology, Michigan State UniversityEast Lansing, MI, USA

**Keywords:** anterograde signaling, light signaling, nuclear gene expression, plastid gene expression, phytochrome, sigma factor

## Abstract

Light perception by photoreceptors impacts plastid transcription, development, and differentiation. This photoreceptor-dependent activity suggests a mechanism for photoregulation of gene expression in the nucleus and plastid that serves to coordinate expression of critical genes of these two organelles. This coordinate expression is required for proper stoichiometric accumulation of components needed for assembly of plastids, photosynthetic light-harvesting complexes and components such as phytochromes. Chloroplast-targeted sigma factors, which function together with the plastid-encoded RNA polymerase to regulate expression of plastid-encoded genes, and nuclear-encoded plastid development factors, such as GLK1 and GLK2, are targets of phytochrome regulation. Such phytochrome-dependent functions are hypothesized to allow light-dependent regulation, and feasibly tuning, of plastid components and function in response to changes in the external environment, which directly affects photosynthesis and the potential for light-induced damage. When the size and protein composition of the light-harvesting complexes are not tuned to the external environment, imbalances in electron transport can impact the cellular redox state and cause cellular damage. We show that phytochromes specifically regulate the expression of multiple factors that function to modulate plastid transcription and, thus, provide a paradigm for coordinate expression of the nuclear and plastid genomes in response to changes in external light conditions. As phytochromes respond to changes in the prevalent wavelengths of light and light intensity, we propose that specific phytochrome-dependent molecular mechanisms are used during light-dependent signaling between the nucleus and chloroplast during photomorphogenesis to coordinate chloroplast development with plant developmental stage and the external environment.

## Introduction

The involvement of regulatory factors or transcriptional regulators in organismal responses to environmental signals is well known. However, specific information about the distinct factors involved is limited in many cases. For example, light quality and quantity are known to stimulate proplastid or dark-prevalent etioplast to light-dependent chloroplast transitions and the expression of many nuclear-encoded, chloroplast-targeted genes (Pogson and Albrecht, 2011). The general importance for a role of some photoreceptors in this process is known. Early studies demonstrated a role for phytochrome-mediated detection of distinct wavelengths in the light-dependent regulation of chloroplast development (Wellburn and Wellburn, 1973). Additional studies demonstrated that both blue light-responsive cryptochrome and red/far-red light-responsive phytochrome photoreceptors perceive light and stimulate plastid development (Thum et al., 2001), and phytochromes are involved in regulating chloroplast gene transcription in mature leaves (Chun et al., 2001). Consistent with these observations, phytochrome-deficient mutants, including *phyB* (Reed et al., 1994) and chromophore-deficient *hy1* and *hy2* mutants (Chory et al., 1989), exhibit defects in chloroplast development and/or differentiation. However, insights into the identity and functions of specific photoreceptor-dependent effectors that impact chloroplast development and function are limited.

Functional plastid development depends upon tight regulation of the expression of nuclear-encoded and plastid genome-encoded photosynthetic genes in the proper stoichiometry, together with the synthesis of photosynthetic pigments (Pogson and Albrecht, 2011). A limited number of factors known to function downstream of the photoreceptors in this process have been identified, including two transcription factors linked to phytochrome function, i.e., PIF1 and PIF3, that have been shown to function as regulators of light-dependent chloroplast development (Monte et al., 2004; Moon et al., 2008; Stephenson et al., 2009). Other transcription factors previously shown to impact the transcription of photosynthesis genes, and thereby chloroplast development, include nuclear Golden2-like (GLK) factors, i.e., GLK1 and GLK2 (Waters et al., 2008), and two plastid-targeted sigma factor (SIG) proteins, i.e., SIG2 and SIG6 (Kanamaru et al., 2001; Ishizaki et al., 2005). GLKs regulate the expression of nuclear-encoded photosynthetic genes (Waters et al., 2008, 2009). SIGs serve as promoter specificity factors that regulate the activity of the plastid-encoded RNA polymerase or PEP (Kanamaru et al., 1999; Hanaoka et al., 2003), which serves to drive expression of genes encoding photosynthetic proteins (Jarvis and López-Juez, 2013).

The expression of *GLK* (Fitter et al., 2002) and *SIG* (Isono et al., 1997; Tsunoyama et al., 2002; Privat et al., 2003; Ishizaki et al., 2005) genes is light-dependent. Notably, *GLK1* and *GLK2* exhibit distinctions, i.e., *GLK1* is primarily regulated by light, whereas *GLK2* appears to be regulated both by light and diurnal cues (Fitter et al., 2002). Furthermore, GLK genes have been shown to have a role in acclimation to light intensity (Waters et al., 2009). The only prior reported connection of these genes to a specific photoreceptor was the regulation of *SIG5* primarily by cryptochromes (Onda et al., 2008) and our recent connection of phytochromes to the regulation of *SIG2* expression (Oh and Montgomery, 2013). The photoreceptor-dependent regulation of such factors is presumed to be critical for “adjusting” or matching chloroplast development and composition to the external environment and to integrating this process with the organismal energy budget.

Plastid biogenesis and development are coordinated with the external environment to optimize plastid-dependent processes such as photosynthesis (Pogson and Albrecht, 2011). Coordinating plastid development with external light impacts the utilization of light for the production of chemical energy during photosynthesis and/or the limitation of photodamage/photoprotection in plants (Pogson and Albrecht, 2011). A failure to coordinate the composition and size of the light-harvesting complexes with the environment can result in the generation of damaging reactive oxygen species. Maintaining the proper stoichiometry of nuclear proteins, plastid proteins, chlorophylls, and carotenoids is critical for assembly of functional photosynthetic and photoprotective complexes in plastids. Thus, factors which coordinate expression of nuclear- and plastid-encoded components of the light-harvesting complexes must have central roles in organismal light responses and photoregulation of chloroplast development. Yet little insight has been gained into the molecular nature of factors that serve as central components of the light-dependent mechanism(s) utilized to coordinate transcription of nuclear- and chloroplast-encoded genes.

Based on recent results which demonstrated that phytochromes regulate the accumulation of photosynthetic proteins encoded by genes from both the nuclear genome and the chloroplast genome (Oh and Montgomery, 2011) and the expression of *SIG2* that encodes a chloroplast transcriptional regulator (Oh and Montgomery, 2013), we investigated the role of phytochromes in regulating other factors that affect plastid development and/or transcription. We sought to elucidate the phytochrome-dependent photoregulation of anterograde signaling between the nucleus and plastid. We determined that phytochromes regulate the expression of another chloroplast transcriptional regulator, SIG6, and a suite of other regulatory and developmental factors that impact plastid transcription and development. These results provide insights into the molecular basis of the central role of phytochromes in coordinating gene expression in the nucleus and chloroplasts during photomorphogenesis. Furthermore, these findings support a hypothesis that phytochromes serve as central integrators of information about the external light environment over time and space to allow plants to finely coordinate plastid function to optimize light capture for photosynthesis, while simultaneously minimizing the potential for light-associated damage and phototoxicity.

## Materials and methods

### Experimental organism and growth conditions

Transgenic *BVR* lines, i.e., 35S::pBVR3 (Montgomery et al., 1999) and CAB3::pBVR2 (Warnasooriya and Montgomery, 2009), and T-DNA insertion mutants, i.e., *phyA* (Mayfield et al., 2007; Ruckle et al., 2007), *phyB* (Mayfield et al., 2007; Ruckle et al., 2007), and double mutant *phyAphyB* (Oh and Montgomery, 2013), were previously described. Sterilized seeds were planted and seedlings grown on MS medium containing 1% (w/v) sucrose and 0.7% (w/v) Phytoblend agar (Caisson Labs, UT) at 22°C for 7 days under the indicated light condition as previously described (Oh and Montgomery, 2013). Light sources utilized for far-red (FR; λ max ~735 nm), red (R; λmax ~670 nm), and white (W) light were described previously (Warnasooriya and Montgomery, 2009). Fluence rates of R, and W were measured using a LI-250A Light Meter (LI-COR) connected to a LI-COR quantum sensor and for FR using a StellarNet EPP2000 spectroradiometer (Apogee Instruments).

### Total RNA extraction and qRT-PCR analysis

RNA samples were extracted from 7-days-old whole seedlings grown under continuous darkness (Dc), continuous FR (FRc) illumination (5 μmol m^−2^s^−1^), continuous R (Rc) illumination (50 μmol m^−2^s^−1^), or white (W; 100 μmol m^−2^s^−1^) using the RNeasy® Plant Minikit (Qiagen, CA) as previously described (Oh and Montgomery, 2013). Quantitative RT-PCR (qRT-PCR) was performed essentially as described previously (Oh and Montgomery, 2013). Briefly, cDNA was synthesized using total RNA (100 ng) and random primers using a Reverse Transcription System (Promega, WI) by following the manufacturer's instructions. The cDNA was then mixed with Fast SYBR® Green Master Mix (Applied Biosystems) and qPCR performed in three technical and three biological replicates using an ABI 7500 Fast Real-Time PCR System (Applied Biosystems). *UBC21* was used as a normalization standard in all qRT-PCR experiments. The primers used for qRT-PCR are indicated in Table 1.

**Table 1:** **Sequence of primers used in this study**.

**AGI number**	**Forward primer sequence (5′-3′)**	**Reverse primer sequence (5′-3′)**	**Purpose**
*At2g36990 (SIG6)*	ctctggagaggaggcagtttg	gccggcaatttcgtttcagat	qRT-PCR analysis
*At2g20570 (GLK1)*	tcattttaggcccctgcatgt	ggattaggcatggcggtagaa	qRT-PCR analysis
*At5g44190 (GLK2)*	aacctaaggtggattggacgc	tttccaagattcgagacggca	qRT-PCR analysis
*At5g25760 (UBC21)*	caaatggaccgctcttatcaaag	ctgaaaaacaccgccttcgt	qRT-PCR analysis

## Results

### Phytochromes impact the expression of multiple nuclear-encoded genes encoding chloroplast-targeted sigma factors

**Figure 1:**
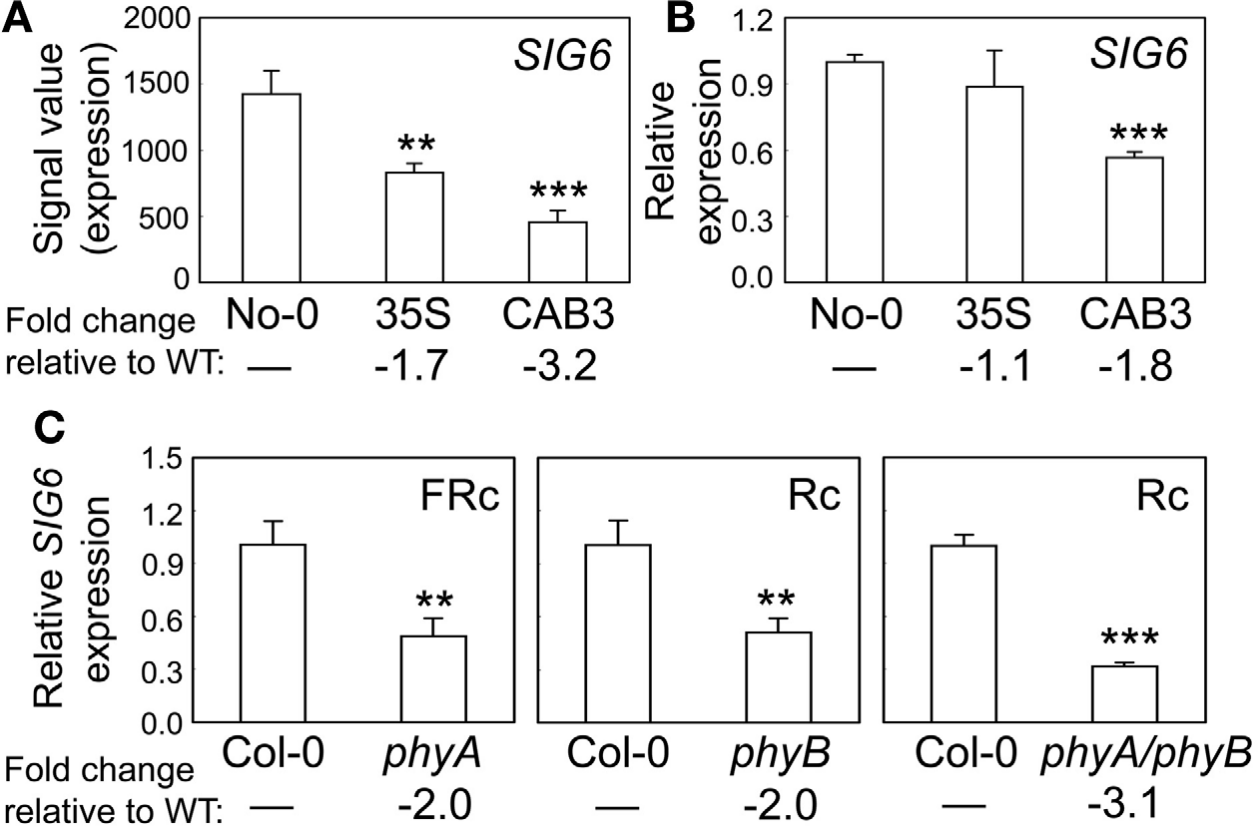
**Phytochrome-dependent regulation of *SIG6* expression. (A)** Expression levels (signal value) of *SIG6* in No-0 wild-type (WT), 35S::pBVR3 (35S), and CAB3::pBVR2 (CAB3) under continuous far-red (FRc) are shown (±*SD*, *n* = 3). Signal value indicates signal intensity on the ATH1 array as calculated by Affymetrix Microarray Suite (MAS). **(B)** Validation of microarray data for *SIG6* using quantitative RT-PCR (qRT-PCR) analysis. Relative *SIG6* expression level compared to *UBC21* is shown (±*SD*, *n* = 3). **(C)** qRT-PCR analysis of *SIG6* expression in Col-0 WT, *phyA* (SALK_014575), *phyB* (SALK_022035), or *phyA/phyB* double mutant seedlings under FRc or continuous red (Rc). Relative *SIG6* expression level compared to *UBC21* is shown (±*SD*, *n* = 3). Statistics: Unpaired, two-tailed Student's *t*-test comparing mutants or transgenic lines to WT, ^**^p < 0.01, ^***^p < 0.005.

We previously demonstrated that the expression of chloroplast-targeted transcriptional regulator *SIG2* is regulated by phytochromes (Oh and Montgomery, 2013). SIG2 is one of six Arabidopsis sigma factors targeted to the chloroplast, whose activity regulates the plastid-encoded RNA polymerase, or PEP (Kanamaru et al., 1999; Allison, 2000). These factors generally serve as promoter-specificity factors (Hanaoka et al., 2003). The level of *SIG6* mRNA also is phytochrome dependent, as its accumulation was significantly lower in a transgenic Arabidopsis line depleted of mesophyll-localized phytochromes through CAB3-promoter-driven expression of a gene encoding a phytochrome chromophore-degrading enzyme biliverdin reductase (BVR) (Figure 1A). As observed for *SIG2* mRNA accumulation (Oh and Montgomery, 2013), we confirmed the downregulation of *SIG6* expression by qRT-PCR in lines depleted of mesophyll-localized phytochromes relative to WT (Figure 1B). We also demonstrate roles for phyA and phyB in the photoregulation of *SIG6* expression by assessing accumulation of *SIG6* mRNA in *phyA*, *phyB*, and *phyAphyB* mutants. *SIG6* was downregulated by ~2-fold in either single *phy* mutants or by 3-fold in the *phyAphyB* double mutant relative to WT (Figure 1C).

**Figure 2:**
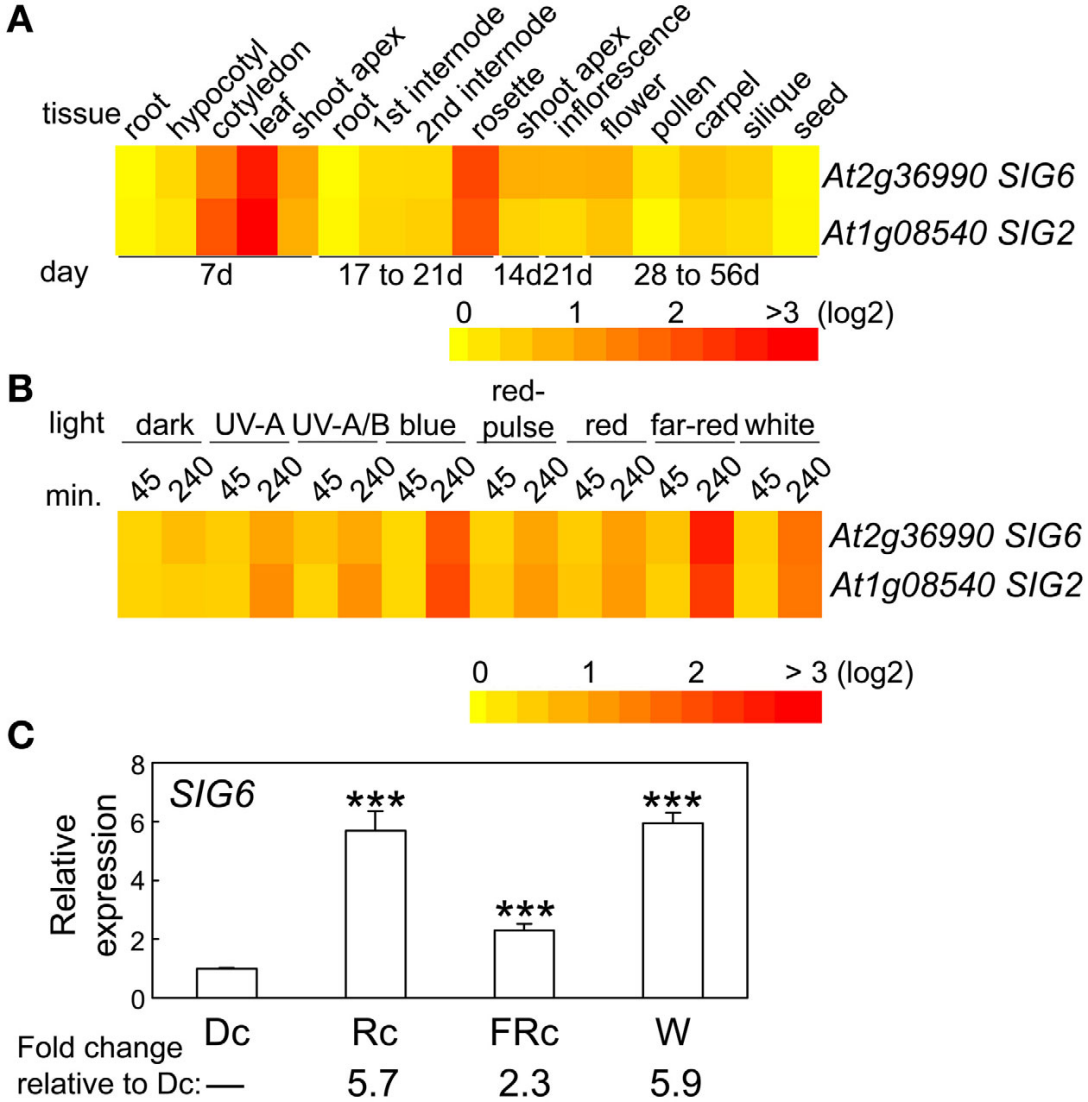
**Expression of *SIG6* in different tissues and light conditions. (A)** Heat map showing the expression of *SIG6* in different tissues indicating mean-normalized values of Col-0 WT from AtGenExpress expression library (www.weigelworld.org) and BAR Heatmapper *Plus* (bar.utoronto.ca). **(B)** Heat map showing the expression of *SIG6* in different light conditions for aerial parts (hypocotyl and cotyledons) of 4-days-old Col-0 WT seedlings grown on MS medium treated with different light for either 45 or 240 min. **(C)** qRT-PCR analysis of *SIG6* expression in Col-0 WT under dark (Dc), Rc, FRc, or white (W). Relative *SIG6* expression level compared to *UBC21* is shown (±*SD*, *n* = 3). Statistics: Unpaired, two-tailed Student's *t*-test comparing different growth conditions ^***^p < 0.005.

The expression of *SIG6* in regards to developmental stage and tissue specificity is very similar to that of *SIG2* (Oh and Montgomery, 2013), i.e., highest expression for *SIG6* was observed in cotyledons, young leaf tissue, and adult rosette leaves (Figure 2A). Light regulation of *SIG6* expression was also similar to *SIG2* (Figure 2B; Oh and Montgomery, 2013). For 7-days-old seedlings, the highest accumulation of *SIG6* mRNA occurred under Rc and W illumination (Figure 2C), again very similar to what was reported for *SIG2* (Oh and Montgomery, 2013).

Although SIG2 and SIG6 have been shown previously to have the most significant effect on chlorophyll accumulation and plastid development among SIG family members (Kanamaru et al., 2001; Ishizaki et al., 2005), we assessed the impact of functional phytochromes on the expression of the remaining SIG family members. The expression of additional *SIG* genes was also impacted by mesophyll phytochrome depletion (Table 2). *SIG1*, *SIG3*, and *SIG4* were downregulated by 2.2-, 3.7-, and 2.4-fold, respectively, in the CAB3::pBVR2 line relative to WT (Table 2). *SIG5* was less impacted than other *SIG* genes for the FRc conditions under which we grew plants, i.e., it was downregulated by only 1.6-fold (Table 2). However, expression of the *SIG5* gene has been previously reported to be mostly regulated by blue light (Tsunoyama et al., 2002, 2004; Onda et al., 2008). The blue light effects on the expression of SIG5-dependent chloroplast genes were characterized as regulated by both cryptochromes and phytochrome A (Thum et al., 2001; Onda et al., 2008).

**Table 2:** **Known light signaling and chloroplast development genes**.

**Gene name**	**AGI No**.	**Average signal value^a^ (expression)**			**Fold change CAB3:WT^b^**
		**WT**	**35S**	**CAB3**	
*SIG1*	*AT1G64860*	1323 ± 201	854 ± 139	578 ± 21	−2.2
*SIG2*	*AT1G08540*	1198 ± 208	738 ± 270	553 ± 156	−2.2
*SIG3*	*AT3G53920*	715 ± 142	386 ± 80	194 ± 29	−3.7
*SIG4*	*AT5G13730*	314 ± 73	235 ± 85	132 ± 18	−2.4
*SIG5*	*AT5G24120*	643 ± 209	442 ± 43	401 ± 49	−1.6
*SIG6*	*AT2G36990*	1426 ± 176	828 ± 70	441 ± 82	−3.2
*PHYA*	*AT1G09570*	3579 ± 181	10220 ± 1849	8248 ± 291	+2.3
*PHYB*	*AT2G18790*	1570 ± 292	1255 ± 153	1321 ± 95	−1.2
*PHYC*	*AT5G35840*	233 ± 26	336 ± 86	328 ± 35	+1.4
*PHYD*	*AT4G16250*	259 ± 40	246 ± 42	266 ± 39	*na*
*PHYE*	*AT4G18130*	233 ± 35	223 ± 78	187 ± 29	−1.2
*PIF1*	*AT2G20180*	463 ± 61	403 ± 49	554 ± 94	+1.2
*PIF3*	*AT1G09530*	231 ± 23	341 ± 108	301 ± 29	+1.3
*PIF4*	*AT2G43010*	577 ± 39	562 ± 72	624 ± 90	+1.1
*GLK1*	*AT2G20570*	285 ± 71	246 ± 29	203 ± 39	−1.4
*GLK2*	*AT5G44190*	1275 ± 181	898 ± 76	612 ± 31	−2.1
*PRDA1*	*AT5G48470*	273 ± 42	146 ± 34	83 ± 29	−3.3
*BSM/RUG2*	*AT4G02990*	617 ± 99	371 ± 34	202 ± 7	−3.1
*DG1*	*AT5G67570*	39 ± 9	53 ± 42	36 ± 17	−1.1
*SG1*	*AT3G18420*	358 ± 86	279 ± 57	286 ± 35	−1.2
*GUN1*	*AT2G31400*	3136 ± 75	2300 ± 285	2163 ± 250	−1.4
*HY2*	*AT3G09150*	205 ± 49	113 ± 13	104 ± 31	−2.0

a *Expression (average signal value) of genes in No-0 wild-type (WT), 35S::pBVR3 (35S), and CAB3::pBVR2 (CAB3) under FRc are indicated (±SD, n = 3), as described previously (Oh et al., 2013). Signal value indicates signal intensity on the ATH1 array as computed by Affymetrix Microarray Suite (MAS). Full microarray data set was previously published (Oh et al., 2013)*.

b *-, expression is lower in CAB3 line compared to WT; +, expression is higher in CAB3 line compared to WT; na, no significant difference between expression in CAB3 line compared to WT*.

### Phytochromes impact the expression of nuclear *GLK* genes, which regulate chloroplast development

**Figure 3:**
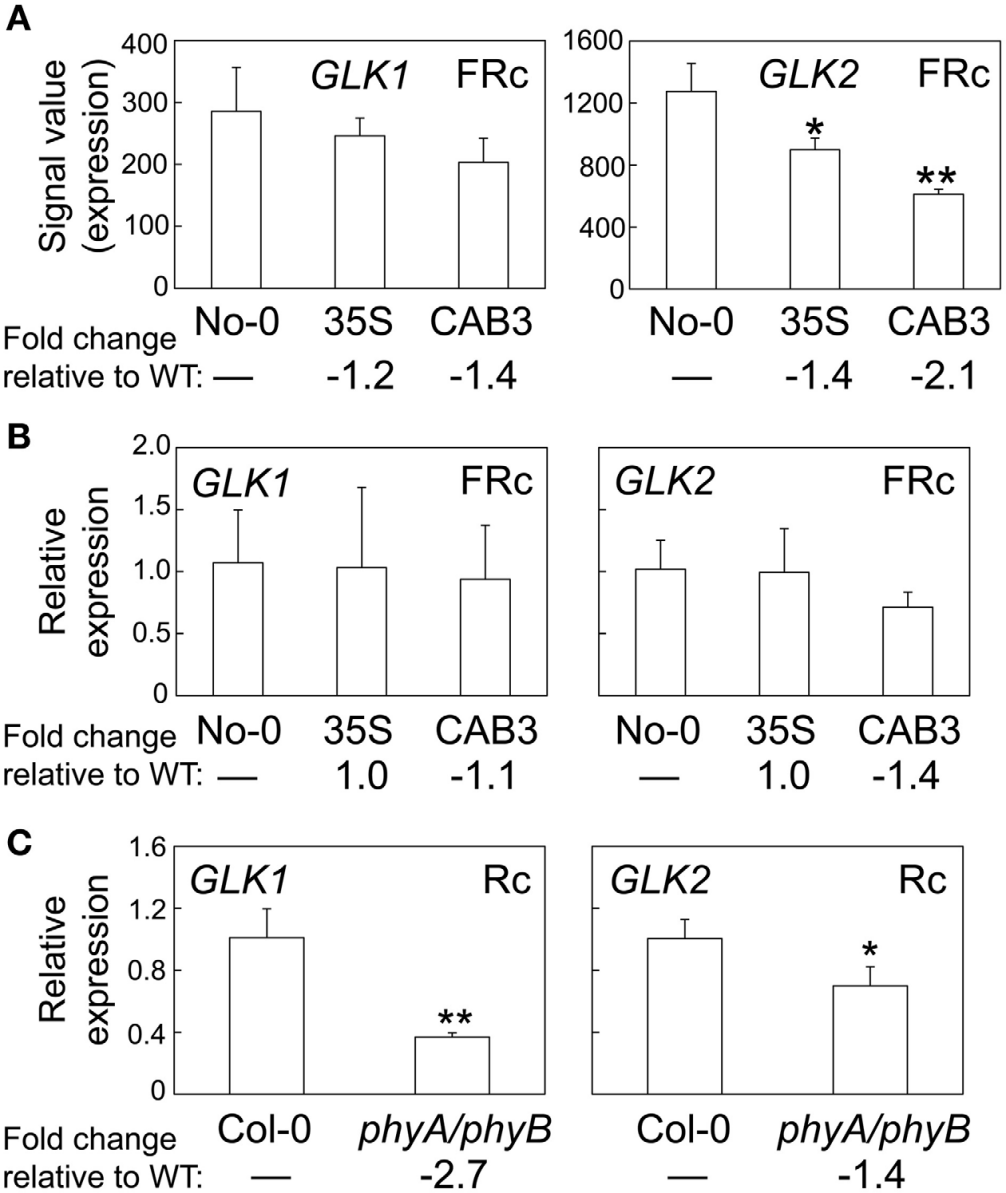
**Phytochrome-dependent regulation of *GLK1* and *GLK2* expression. (A)** Expression levels (signal value) of *GLK1* or *GLK2* in No-0 WT, 35S, and CAB3 under FRc are shown (±*SD*, *n* = 3). **(B)** qRT-PCR validation of microarray data for *GLK1* or *GLK2* under FRc. Relative *GLK1* or *GLK2* expression level compared to *UBC21* is shown (±*SD*, *n* = 3). **(C)** qRT-PCR quantification of *GLK1* or *GLK2* expression in Col-0 WT or *phyA/phyB* double mutant seedlings under Rc. Relative *GLK1* or *GLK2* expression level compared to *UBC21* is shown (±*SD*, *n* = 3). Statistics: Unpaired, two-tailed Student's *t*-test comparing mutants or transgenic lines to WT, ^*^p < 0.05, ^**^p < 0.01.

A number of genes previously demonstrated to be involved in chloroplast development also were identified as misregulated in our microarray analysis assessing lines depleted of phytochromes (Table 2). There were minimal effects on the expression of *PIF1* and *PIF3* under FRc. However, other genes previously shown to impact plastid development were downregulated when mesophyll phytochromes were depleted. Specifically, we identified misregulation of the expression of two nuclear transcription factors, i.e., *Golden2-like 1* (*GLK1*) and *Golden2-like 2* (*GLK2*) (Table 2), which have been previously shown to impact expression of nuclear photosynthetic genes linked to chloroplast development (Waters et al., 2008, 2009). In agreement with our observed link to phytochrome activity, prior analysis of publicly available microarray data indicated that expression of *GLK1* and *GLK2* is promoted by red and blue light (Waters et al., 2008). Expression of *GLK2* was significantly downregulated in lines depleted of mesophyll-localized phytochromes (Figure 3A; Oh and Montgomery, 2013). A greater impact on *GLK2* expression than *GLK1* was confirmed by qRT-PCR analyses (Figure 3B). Notably, both *GLK1* and *GLK2* expression was significantly downregulated in a *phyAphyB* double mutant under Rc light (Figure 3C), definitively indicating that expression of both *GLK* genes is impacted by phytochromes *in vivo*.

**Figure 4:**
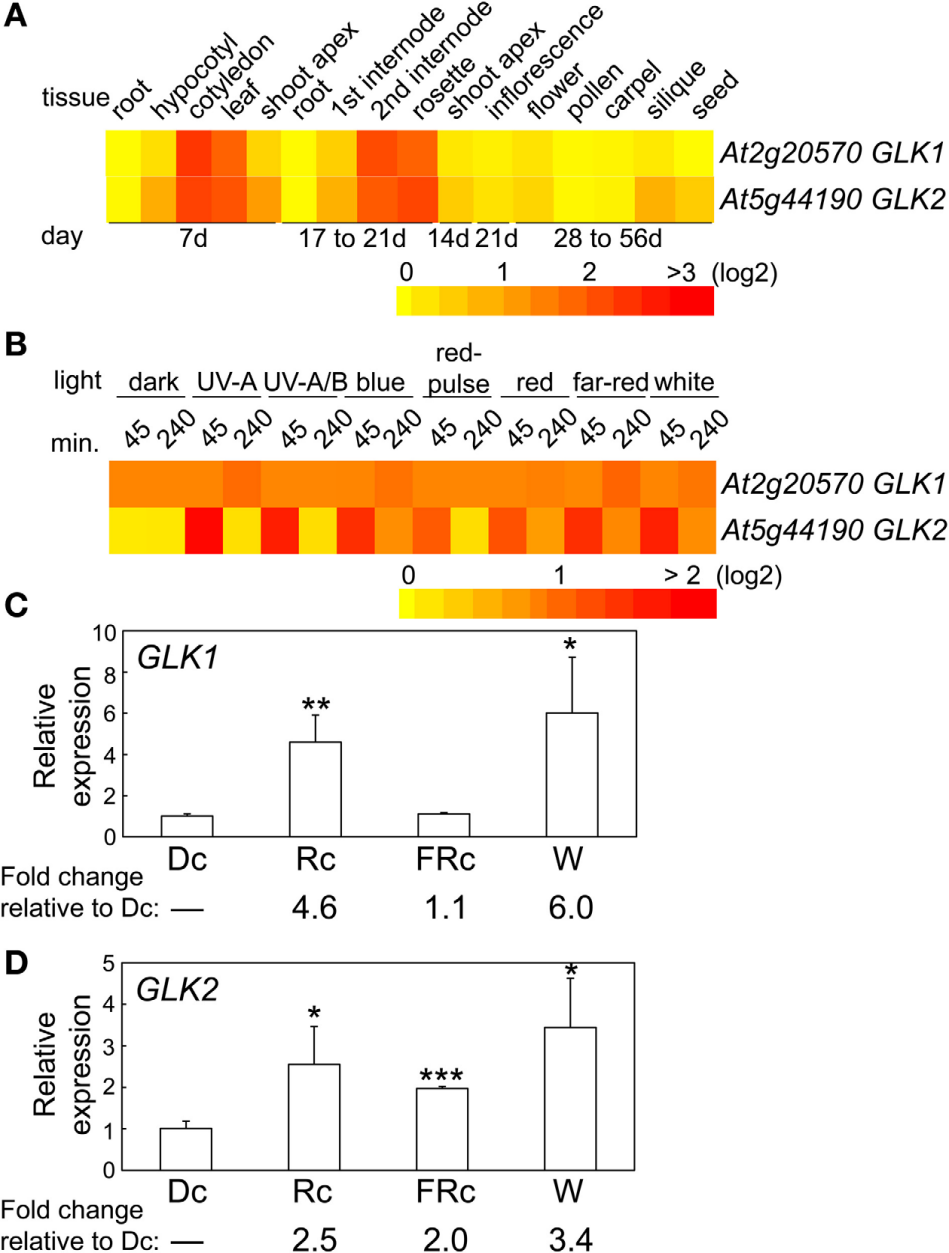
**Expression of *GLK1* and *GLK2* in different tissues and light conditions. (A)** Heat map showing the expression of *GLK1* or *GLK2* in different tissues indicating mean-normalized values of Col-0 WT from AtGenExpress expression library (www.weigelworld.org) and BAR Heatmapper *Plus* (bar.utoronto.ca). **(B)** Heat map showing the expression of *GLK1* or *GLK2* in different light conditions for aerial parts (hypocotyl and cotyledons) of 4-days-old Col-0 WT seedlings grown on MS medium treated with different light for either 45 or 240 min. qRT-PCR analysis of **(C)** *GLK1* or **(D)** *GLK2* expression in Col-0 WT under dark (Dc), Rc, FRc, or white (W). Relative *GLK1* or *GLK2* expression level compared to *UBC21* is shown (±*SD*, *n* = 3). Statistics: Unpaired, two-tailed Student's *t*-test comparing different growth conditions ^*^p < 0.05, ^**^p < 0.01, ^***^p < 0.005.

The two *GLK* genes are predicted to have a similar distribution of mRNA accumulation in different tissues and at different developmental stages (Figure 4A). Both genes are highly expressed in cotyledons, young leaf tissues, adult rosette leaves, and internodes (Figure 4A). However, expression of *GLK2* appears to be more significantly impacted by light than *GLK1* (Figure 4B), at least in the 4-days-old seedlings used for the experiments included in the heat map. We analyzed expression of the two *GLK* genes in 7-days-old seedlings to assess the impact of light on their expression for seedlings of the same age as those used in the microarray analysis in which we noted a phytochrome-dependent regulation of *GLK* expression. In these analyses, we observed a significant impact of Rc and W light on *GLK1* expression, whereas FRc light had no significant effect on *GLK1* expression (Figure 4C). By comparison, *GLK2* was significantly upregulated by Rc, FRc, and W light relative to darkness, although the impact of Rc and W were much more robust than FRc (Figure 4D).

### Phytochromes impact the expression of additional chloroplast development genes

Additional plastid-targeted factors that have been associated with light-dependent regulation of plastid development were impacted by mesophyll-localized phytochrome function. These genes include *PEP-Related Development Arrested 1* (*PRDA1*) and *BELAYA SMERT/RUGOSA2* (*BSM/RUG2*). *PRDA1* is a nuclear genome-encoded gene which encodes a plastid-targeted protein that impacts plastid transcription (Qiao et al., 2013). Co-expression analyses and protein interaction studies indicated that PRDA1 functions in PEP-related chloroplast transcriptional regulation to affect plastid development (Qiao et al., 2013). Notably, a *prda1* mutant exhibits a similar change in plastid gene expression to other PEP mutants, including *sig6* (Qiao et al., 2013). The accumulation of mRNA for *PRDA1* was downregulated by 3.3-fold in lines depleted of mesophyll phytochromes under FRc conditions (Table 2), suggesting that this chloroplast developmental regulator is also a target of phytochrome regulation.

*BSM/RUG2* is a nuclear gene that encodes a plastid-targeted protein of the mitochondrial transcription termination factor (mTERF) family, which impacts plastid gene expression (Babiychuk et al., 2011; Quesada et al., 2011). Expression of *BSM/RUG2* is positively regulated by phytochromes, as the gene is downregulated by 3.1-fold in a mesophyll phytochrome-depleted line (Table 2). The only light condition previously reported to impact *BSM/RUG2* expression was high light (Quesada et al., 2011). Thus, the regulation of expression of this gene by phytochrome may be correlated with the ability of this photoreceptor family to respond to light intensity, in addition to light quality (Fankhauser, 2001).

It should be noted that the impact of phytochrome appears to be specific to some chloroplast development genes, while apparently having no influence on others. Two genes encoding a pentratricopeptide repeat (PPR) protein and a tetratricopeptide protein (TPR) that have been shown to impact chloroplast development show distinct patterns in our array data for lines depleted of mesophyll phytochromes. The expression of PPR-encoding gene *DELAYED GREENING1* (*DG1*) has been shown to be light-dependent and to impact PEP-dependent transcription of chloroplast genes (Chi et al., 2008). TPR-encoding *SLOW GREEN1* (*SG1*) also impacts expression of chloroplast- and nuclear-encoded genes (Hu et al., 2014). However, the expression of *DG1* and *SG1* are not significantly impacted in the CAB3::pBVR2 line relative to WT (Table 2). These results suggest that phytochromes selectively regulate the expression of particular genes which impact chloroplast development and differentiation, while having no direct impact on others. It should be noted, however, that DG1 has been shown to interact with SIG6, and thus DG1 function may be indirectly impacted by phytochromes through the phytochrome-dependent regulation of *SIG6* expression (Chi et al., 2010).

## Discussion

**Figure 5:**
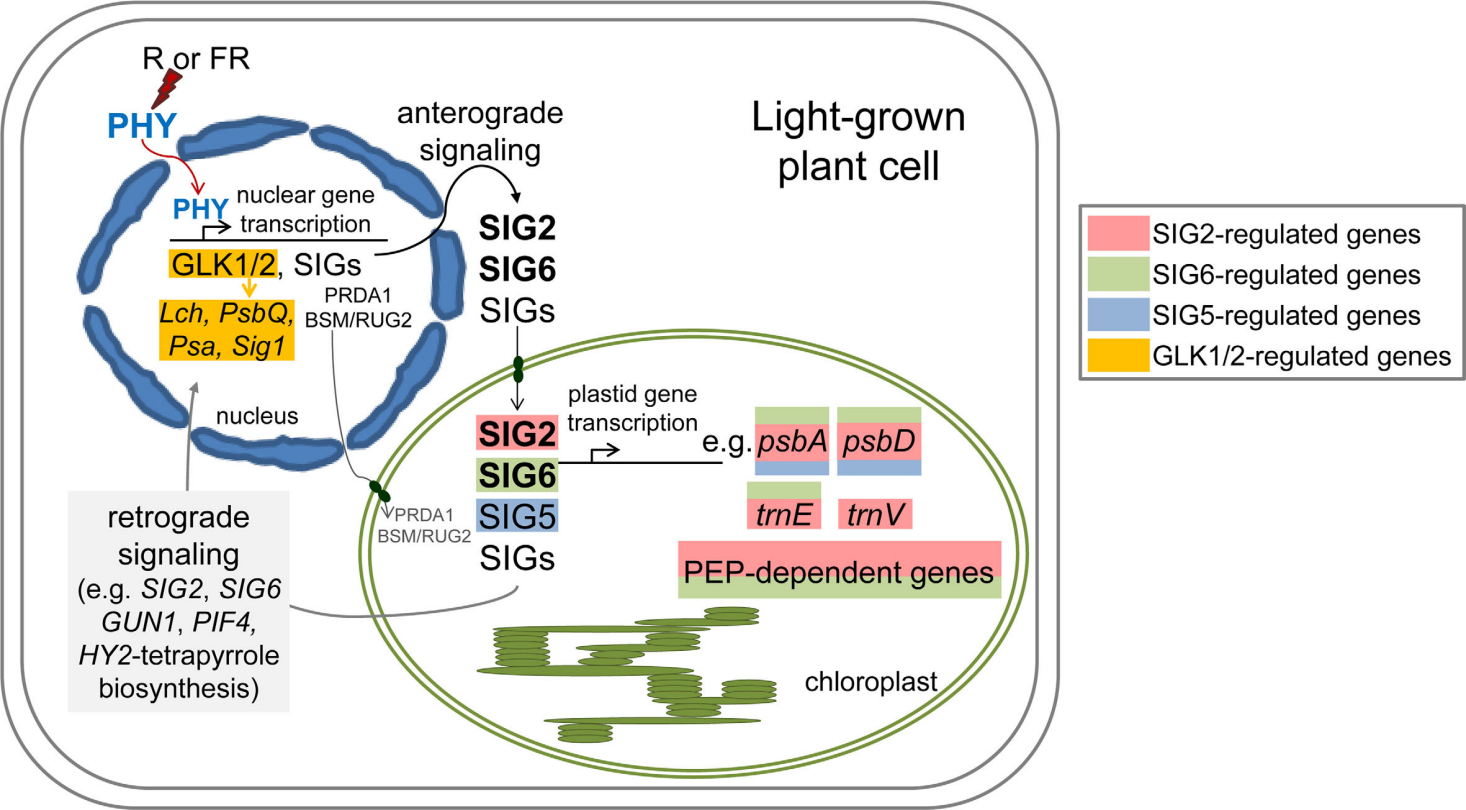
**Model of light- and phytochrome-dependent regulation of anterograde signaling between the nucleus and plastid during chloroplast development and photomorphogenesis**. PEP, Plastid-encoded plastid RNA polymerase; PHY, phytochrome; R, red; FR, far-red.

We have identified a number of nuclear factors and nuclear-encoded, plastid-targeted factors that impact chloroplast development and whose expression is regulated in a light-dependent manner by phytochromes (Figures 1–4; Oh and Montgomery, 2013). The expression of factors centrally involved in the early stages of light-dependent chloroplast development, including SIG2, SIG6, GLK1, and GLK2, is most highly upregulated under red and white light (Figures 1–4; Oh and Montgomery, 2013). Notably, red light was defined very early as the condition under which chloroplast development is most significantly stimulated (Wellburn and Wellburn, 1973). Chlorophyll-deficient phenotypes (Kanamaru et al., 2001; Ishizaki et al., 2005; Waters et al., 2008, 2009; Babiychuk et al., 2011; Quesada et al., 2011; Oh and Montgomery, 2013; Oh et al., 2013; Qiao et al., 2013; Woodson et al., 2013) or high light-sensitive phenotypes (Warnasooriya and Montgomery, 2009; Waters et al., 2009; Quesada et al., 2011) have been noted for lines with attenuated or complete knockdown of the expression of phytochrome-regulated genes described here. These findings support solid hypotheses about the mechanistic bases of interactions between photomorphogenesis and chloroplast development at the molecular level. We hypothesize that phytochromes regulate a number of factors that serve as a central part of the mechanism to coordinate transcription of nuclear genes and chloroplast genes to ensure the correct stoichiometric production of components of the photosynthetic light harvesting complexes and the production of components, such as phytochromes themselves, which depend upon products from these two organelles (Figure 5).

It was previously suggested that such light- and photoreceptor-dependent regulation of *GLK* expression may serve as a mechanism for adjusting photosynthesis in cells with environmental inputs (Waters et al., 2008). Notably, the phenotype of a double *glk1glk2* mutant is distinct from BVR or *sig* phenotypes, as the small and large subunits of Rubisco accumulate to normal levels in a *glk1glk2* mutant (Waters et al., 2009), but are disrupted in lines depleted of mesophyll phytochromes (Oh and Montgomery, 2011) and *sig2* mutants (Kanamaru et al., 2001). Also, there is a defective etioplast phenotype for the double *glk1glk2* mutant (Waters et al., 2009) and for the *pif1pif3* double mutant (Stephenson et al., 2009), but no apparent dark-dependent phenotype for *sig2* (Kanamaru et al., 2001) or *sig6* mutants (Ishizaki et al., 2005). Interestingly, a role for *SIG2* in coordinating expression of photosynthetic light-harvesting genes between nuclear and chloroplast genomes also has recently been observed in the red alga *Cyanidioschyzon merolae* (Fujii et al., 2013), extending a role of this protein in light-dependent anterograde signaling between the nucleus and chloroplasts beyond higher plants. *SIG2* expression in *C. merolae* is also light-induced (Fujii et al., 2013), suggesting that a similar photoreceptor-dependent mechanism for regulating SIG accumulation (Figure 5), and thus coordination of light-harvesting complex assembly, may be employed in *C. merolae*. It has been suggested that photoregulation of the expression of SIG factors may also contribute to the observed light-dependent activation of some PEP-associated, plant-specific factors, including plastid transcriptionally active chromosome (pTAC) components, that impact plastid transcription (Yagi et al., 2012). Such regulatory mechanisms are hypothesized to allow modulation of chloroplast development in response to adverse environmental conditions (Yagi and Shiina, 2014).

Although not reported for all of the genes described to function in phytochrome-dependent anterograde signaling between the nucleus and plastids, SIG2 and SIG6 also have been reported to function in retrograde signaling, in which the functional state of plastids can feed back to impact expression of nuclear-encoded photosynthetic genes (Woodson et al., 2013; Figure 5). Thus, SIG2 and SIG6 have central roles in the establishment of plastid function during photomorphogenesis and in surveillance of plastid function during growth. Whether other proteins controlled by phytochromes during anterograde signaling have similar dual roles in retrograde signaling is not yet well understood.

Phytochrome-dependent regulation of anterograde signaling between the nucleus and plastids appears to be important for both development and differentiation of chloroplasts, as well as fine tuning of the organelles in response to fluctuations in environmental parameters such as light intensity. These results provide significant new information about the molecular mechanisms used for light-dependent anterograde signaling between the nucleus and chloroplast during photomorphogenesis. Additional studies to identify and characterize the distinct photoreceptors and photoreceptor-dependent effectors that impact the full complement of factors that function in the light-dependent coordination of gene expression in the nucleus and chloroplast will be required for a more complete understanding of this vital process.

### Conflict of interest statement

The authors declare that the research was conducted in the absence of any commercial or financial relationships that could be construed as a potential conflict of interest.
